# Early, Supervised Point-of-Care Ultrasound Training for Internal Medicine Interns: A Quality Improvement Initiative

**DOI:** 10.7759/cureus.103476

**Published:** 2026-02-12

**Authors:** Thar Sann Oo, Nikola Tanasijevic, Damion Hunter, Ashot Batikyan, Minas Sakellakis, Michail Spanos, Mallika Pradhan

**Affiliations:** 1 Department of Internal Medicine, Albert Einstein College of Medicine, Jacobi Medical Center/North Central Bronx Hospital, New York, USA; 2 Department of Pulmonary and Critical Care Medicine, Albert Einstein College of Medicine, Jacobi Medical Center/North Central Bronx Hospital, New York, USA

**Keywords:** graduate medical education, internal medicine residency, medical education, point of care ultrasound, quality improvement, residency training, ultrasound education, ultrasound training

## Abstract

Background and objective

Point-of-care ultrasound (POCUS) is increasingly being integrated into inpatient internal medicine; however, early residency training remains inconsistent. Objective evaluations of structured early-intern POCUS curricula that assess both knowledge and technical competency are limited. This study aimed to address this shortcoming through a quality improvement initiative.

Methods

We conducted a six-month quality improvement initiative within an internal medicine residency program (July-December 2023). Twenty interns participated in an early, structured POCUS curriculum consisting of didactics, supervised bedside training, and longitudinal reinforcement. Knowledge was assessed using a 15-question multiple-choice examination administered pre- and post-intervention. Practical competency was evaluated post-intervention using a standardized 30-point skills rubric. Paired knowledge outcomes were compared using the exact McNemar’s test.

Results

At baseline, 10% (2/20) of participants met the passing threshold on the knowledge examination. Post-intervention, 95% (19/20) achieved a passing score (p < 0.001). Notably, 90% (18/20) passed the composite practical skills assessment. While 87% demonstrated proficiency in landmark identification and 65% achieved adequate overall image acquisition, performance was lower in advanced applications, including inferior vena cava measurement (35%) and aortic valve visualization (45%).

Conclusions

An early, supervised POCUS curriculum was associated with significant improvements in intern knowledge and high rates of technical competency. Descriptive analysis identified predictable limitations in advanced cardiac and inferior vena cava assessments, which informed targeted curricular refinement. Further research is required to evaluate long-term retention and clinical impact.

## Introduction

Point-of-care ultrasound (POCUS) is becoming an increasingly integral component of bedside evaluation in inpatient internal medicine, with established utility in cardiopulmonary assessment, volume status estimation, and procedural guidance [1,2]. National professional organizations, including the Society of Hospital Medicine and the Alliance of Academic Internal Medicine, have formally endorsed POCUS as a core competency for hospital-based internists and internal medicine trainees [1,3].

Despite national endorsement, implementation of POCUS training within internal medicine residency programs remains inconsistent. National surveys demonstrate substantial variability in curricular structure, instructional intensity, and assessment methods, resulting in heterogeneous resident confidence and skill levels, particularly for cardiac and hemodynamic applications central to inpatient care [4,5]. Many incoming interns begin residency with minimal prior ultrasound exposure, and delayed or optional training may limit early clinical utilization and slow foundational skill acquisition during the intern year [4,6].

Early, structured, and supervised POCUS curricula have been proposed to normalize ultrasound use and accelerate competency development in internal medicine trainees. While longitudinal curricula have been associated with improved intern confidence and bedside ultrasound utilization, objective evaluations of early intern-level curricula assessing both knowledge acquisition and practical technical competency remain limited [5,6]. In light of this, we aimed to evaluate whether an early, six-month longitudinal POCUS curriculum implemented at the start of intern year was associated with improvements in intern knowledge and practical scanning competency.

## Materials and methods

Study design and setting

We conducted a six-month quality improvement initiative within an internal medicine residency program at North Central Bronx Hospital (Bronx, NY, USA). The initiative spanned from July 2023 through December 2023, corresponding to the first six months of the academic intern year. The project was reviewed and designated as a quality improvement initiative and was exempt from institutional review board oversight.

Participants

All first-year internal medicine interns entering residency during the study period were eligible to participate. Twenty interns were included in the analysis.

Intervention

The intervention consisted of didactic instruction, supervised hands-on POCUS training, and structured documentation using standardized Epic electronic health record smartphrases, consistent with prior descriptions of real-time POCUS documentation in the electronic health record [7], as outlined in Table 1. Didactic instruction covered ultrasound fundamentals, probe selection, image optimization, and core inpatient applications relevant to internal medicine.

**Table 1:** Standardized Epic electronic health record smartphrase for POCUS documentation POCUS: point-of-care ultrasound; PLAX: parasternal long-axis; PSAX: parasternal short-axis; IVC: inferior vena cava

Ultrasound domain	Assessment component	Recorded elements
Lung ultrasound	B-lines	Presence or absence; anatomic zone (upper/middle/lower; anterior/posterior)
	Pleural effusion	Presence or absence
	Lung sliding	Present or absent
	Air bronchograms	Present or absent
Cardiac ultrasound	PLAX	Left ventricular systolic function (normal/reduced/equivocal); right ventricular size (normal/dilated); left ventricular size (normal/dilated)
	PSAX	Right and left ventricular size and qualitative function
	Apical four-chamber view	Biventricular size and systolic function
	Subcostal view	Qualitative ventricular assessment
IVC	Maximal diameter	< 2.1 cm or ≥ 2.1 cm
	Collapsibility (with sniff)	> 50% or ≤ 50%

An introductory eight-week curriculum was implemented at the start of the academic year, consisting of sequential didactic sessions and supervised in-person bedside training focused on cardiac, lung, and inferior vena cava ultrasound, as outlined in Table 2. Supervised sessions emphasized real-time image acquisition and anatomic landmark identification on volunteer residents under faculty supervision.

**Table 2:** Structure of the eight-week introductory POCUS curriculum POCUS: point-of-care ultrasound; US: ultrasound; A-line: horizontal reverberation artifact; B-line: vertical reverberation artifact; IVC: inferior vena cava; PLAX: parasternal long-axis; PSAX: parasternal short-axis; M-mode: motion mode; PPT: PowerPoint; .phrase: Epic electronic health record smartphrase

Week	Curriculum content
Week 1	Intro POCUS PPT
Week 2	In-person: US basics and nomenclature
Week 3	In-person: bedside lung POCUS (A/B-line)
Week 4	In-person: bedside lung POCUS (effusion, sliding, M-mode)
Week 5	In-person: bedside cardiac POCUS (IVC and subxiphoid)
Week 6	In-person: bedside cardiac POCUS (PLAX, PSAX, apical)
Week 7	In-person: bedside cardiac POCUS (PLAX, PSAX, apical)
Week 8	Documentation: reports & .phrase w/Excel

To ensure educational consistency, hands-on bedside sessions were led by four critical care faculty members with advanced POCUS skills and over three years of clinical experience. These sessions maintained an instructor-to-learner ratio of 1:20. Following the initial eight-week curriculum, longitudinal reinforcement (months two to six) involved bi-weekly supervised sessions performed by either faculty or senior residents. During this period, interns were encouraged to meet a goal of documenting at least two clinical scans per month using the standardized Epic smartphrases.

Quality improvement framework

This initiative followed a Plan-Do-Study-Act (PDSA) framework [8].

Plan

An eight-week POCUS curriculum was implemented at the start of the academic year, including didactic instruction, supervised hands-on training, and Epic-integrated documentation tools. Baseline exposure to POCUS among incoming interns was assessed using a pre-intervention questionnaire, reflecting limited prior familiarity.

Do

A six-month intervention was implemented, including the use of a standardized Epic smartphrase for POCUS documentation and interval reinforcement through supervised hands-on practice in the clinical setting.

Study

Intern-reported POCUS documentation was reviewed longitudinally to inform curricular refinement; however, process measures were not included in formal outcome analysis. At the conclusion of the six-month intervention, knowledge and practical skills were assessed using an objective multiple-choice examination and a composite practical skills assessment.

Act

Curricular refinements were implemented based on identified technical gaps, and the training was permanently integrated into the residency program curriculum.

The assessment process was standardized across pre-intervention and post-intervention phases to ensure objective measurement of knowledge acquisition and technical skill development.

Assessment

Theoretical competency was evaluated using a 15-question True/False multiple-choice questionnaire (MCQ). The same instrument was administered pre-intervention to establish a baseline and post-intervention to measure growth. The questionnaire covered ultrasound physics (e.g., gain, depth), probe maneuvers including fanning, rocking, and rotation, and the sonographic interpretation of specific findings such as the Seashore, Batwing, Curtain, and Barcode signs. A passing score for the knowledge examination was defined as at least 10 out of 15 correct responses (67%).

To minimize assessment bias, practical competency was graded using a standardized 30-point binary rubric (landmark present/absent). Assessors were not blinded to intern identities or pre-intervention knowledge scores. Assessments were conducted on volunteer residents rather than patients to standardize the technical difficulty of image acquisition. Interns were assessed across seven primary ultrasound windows, including cardiac views (parasternal long axis, parasternal short axis, apical four-chamber, and subcostal), the inferior vena cava (diameter and collapsibility), and lung zones (anterior and lateral).

A 30-point standardized rubric was utilized to grade performance based on two domains: landmark identification (correct anatomical probe placement and marker orientation) and image acquisition (successful visualization of specific diagnostic structures, such as the "fish mouth" appearance of the mitral valve in the parasternal short axis or lung sliding in M-mode). The passing threshold for technical competency was set at a composite score of 20 out of 30 points (67%).

Data analysis

Performance outcomes were summarized descriptively using proportions. Window-level performance was analyzed to identify areas of relative strength and technical limitation within the curriculum. Pre-intervention and post-intervention knowledge examination outcomes were compared using the exact McNemar’s test for paired binary data. Statistical significance was set at p < 0.05.

## Results

Twenty interns participated in the initiative. At baseline, 10% (2/20) of participants met the passing threshold on the knowledge examination. Following the intervention, the knowledge examination pass rate significantly improved to 95% (19/20; 95% CI: 75.1, 99.9), representing a statistically significant increase in theoretical proficiency (p<0.001, exact McNemar’s test). Regarding technical competency, 90% (18/20; 95% CI: 68.3, 98.8) of interns successfully passed the composite practical skills examination by achieving the required 20 out of 30 points.

Detailed window-level analysis demonstrated that while 85% (17/20; 95% CI: 62.1, 96.8) of interns achieved proficiency in basic landmark identification, 65% (13/20; 95% CI: 40.8, 84.6) obtained overall adequate image acquisition. Specific technical limitations were most pronounced in advanced applications, with success rates of 35% for inferior vena cava diameter and collapsibility measurement, 45% for aortic valve visualization, and 75% for diaphragmatic visualization. Post-intervention knowledge and practical competency outcomes are summarized in Table 3.

**Table 3:** Pre- and post-intervention knowledge and post-intervention practical competency outcomes among internal medicine interns Values are reported as number (n), percentage (%), and 95% confidence interval (CI). Pre-intervention assessment was performed for the knowledge examination only. Practical skills and window-specific performance were assessed post-intervention only. 95% CIs were calculated using the Wilson Score method to account for the small sample size (N=20) and proportions near 0% or 100%. Paired pre- and post-intervention knowledge examination outcomes were compared using the exact McNemar's test. Statistical significance was defined as p < 0.05

Outcome measure	Pre-intervention, n/N (%) (95% CI)	Post-Intervention, n/N (%) (95% CI)	Statistical test (p-value)
Knowledge examination (MCQ)	2/20 (10%) (2.8, 30.1)	19/20 (95%) (75.1, 99.9)	Exact McNemar's test, p < 0.001
Composite practical skills exam	—	18/20 (90%) (68.3, 98.8)	Not applicable
Technical proficiency			
Basic landmark identification	—	17/20 (87%) (62.1, 96.8)	Not applicable
Overall adequate image acquisition	—	13/20 (63%) (40.8, 84.6)	Not applicable
Window-specific performance			
Diaphragm visualization (lung)	—	15/20 (75%) (50.9, 91.3)	Not applicable
Aortic valve visualization (cardiac)	—	9/20 (45%) (23.1, 68.5)	Not applicable
Inferior vena cava measurement	—	7/20 (35%) (15.4, 59.2)	Not applicable

## Discussion

This quality improvement initiative suggests that an early, supervised POCUS curriculum is a feasible model for intern training and is associated with high post-intervention knowledge and practical competency, although its direct effectiveness compared to other models was not tested. These findings align with national position statements from the Society of Hospital Medicine and the Alliance of Academic Internal Medicine, which advocate for structured POCUS education during internal medicine training [1,3,5]. Prior literature has shown substantial heterogeneity in the timing, scope, and assessment of POCUS training across internal medicine residency programs. Many programs rely on elective, later-stage, or self-directed exposure, which has been associated with inconsistent skill acquisition and limited faculty supervision [4]. Early integration of POCUS training, as demonstrated in this initiative, may normalize ultrasound use as a routine component of inpatient clinical assessment rather than as an advanced or optional skill set.

Comparative studies suggest that early, structured exposure accelerates competency development. Schnobrich et al. demonstrated that a structured internal medicine POCUS curriculum introduced early in residency was feasible and associated with improvements in resident knowledge, technical skills, and confidence compared with informal exposure models [9]. Similarly, longitudinal and multimodal POCUS curricula incorporating simulation and supervised bedside instruction have been shown to improve image acquisition quality and learner performance over time [6,10]. The high post-intervention knowledge and practical skills pass rates observed in this cohort are consistent with these findings.

Window-level descriptive analysis identified predictable technical limitations, particularly in inferior vena cava measurement and advanced cardiac views such as the aortic valve. Prior studies have shown that advanced cardiac windows and quantitative measurements are among the most challenging POCUS applications for internal medicine trainees and typically require greater longitudinal exposure and deliberate practice [2,7]. In contrast, foundational lung and basic cardiac views are more reliably acquired early in training. These findings support a tiered educational strategy that prioritizes core applications early, with progressive advancement rather than uniform expansion of instructional time.

The use of a PDSA framework enabled iterative curricular refinement based on objective performance data. PDSA methodology is well established in educational quality improvement and has been widely applied to curriculum development and evaluation in graduate medical education [8]. This approach allowed targeted adjustments focused on identified technical gaps rather than reliance on learner self-assessment alone.

Limitations

This initiative has several limitations. First, as a single-site study with a small cohort (N=20), our findings lack broad generalizability, and the resulting wide confidence intervals reflect significant statistical imprecision. Second, the study design lacked a contemporaneous control group, which restricts our ability to establish definitive causal links between the curriculum and competency. Third, while baseline knowledge was found to be low, we did not perform a pre-intervention practical assessment; baseline technical competence was assumed rather than measured. Fourth, operational and assessment constraints may have influenced outcomes: the 1:20 instructor-to-learner ratio during initial bedside sessions potentially limited individualized coaching, while the non-mandatory nature of longitudinal scanning goals introduced practice variability.

Furthermore, inter-rater reliability for the practical skills examination was not formally measured, and assessors were not blinded to the participants, creating a potential for assessment bias. Fifth, the 67% passing threshold for both examinations was determined by internal faculty consensus and lacks external validation. Finally, outcomes were assessed only at the conclusion of the six-month intervention; long-term skill retention, diagnostic accuracy, and downstream patient-centered outcomes were not evaluated. Prior literature suggests that without ongoing longitudinal reinforcement, POCUS skills may deteriorate over time [6,10].

Future research directions

Future work should evaluate the durability of early-acquired POCUS skills, the impact of longitudinal reinforcement during later residency years, and the effects on clinical utilization and patient outcomes. Comparative studies examining early versus delayed POCUS curricula across internal medicine programs would further help inform best practices for curriculum design and implementation.

## Conclusions

Our findings show that early, supervised POCUS training was associated with high post-intervention knowledge and practical competency scores among internal medicine interns. While these results are promising for early-internship skill acquisition, further research is required to determine the impact on clinical decision-making and long-term skill retention. Descriptive window-level analysis revealed consistent challenges in advanced cardiac and inferior vena cava assessments, identifying targets for curricular refinement. The curriculum has since been continued and integrated into subsequent intern cohorts to support ongoing skill development and iterative program improvement.
